# Local SAR compression with overestimation control to reduce maximum relative SAR overestimation and improve multi-channel RF array performance

**DOI:** 10.1007/s10334-020-00890-0

**Published:** 2020-09-22

**Authors:** Stephan Orzada, Thomas M. Fiedler, Andreas K. Bitz, Mark E. Ladd, Harald H. Quick

**Affiliations:** 1 Erwin L. Hahn Institute for Magnetic Resonance Imaging, University Duisburg-Essen, Kokereiallee 7, 45141 Essen, Germany; 2 High-Field and Hybrid MR Imaging, University Hospital Essen, 45147 Essen, Germany; 3 Medical Physics in Radiology, German Cancer Research Center (DKFZ), Im Neuenheimer Feld 280, 69120 Heidelberg, Germany; 4 Electromagnetic Theory and Applied Mathematics, Faculty of Electrical Engineering and Information Technology, FH Aachen, University of Applied Sciences, 52066 Aachen, Germany; 5 Faculty of Physics and Astronomy, University of Heidelberg, Im Neuenheimer Feld 226, 69120 Heidelberg, Germany; 6 Faculty of Medicine, University of Heidelberg, Im Neuenheimer Feld 672, 69120 Heidelberg, Germany

**Keywords:** SAR, Vops, VOP compression, MRI, Local SAR

## Abstract

**Objective:**

In local SAR compression algorithms, the overestimation is generally not linearly dependent on actual local SAR. This can lead to large relative overestimation at low actual SAR values, unnecessarily constraining transmit array performance.

**Method:**

Two strategies are proposed to reduce maximum relative overestimation for a given number of VOPs. The first strategy uses an overestimation matrix that roughly approximates actual local SAR; the second strategy uses a small set of pre-calculated VOPs as the overestimation term for the compression.

**Result:**

Comparison with a previous method shows that for a given maximum relative overestimation the number of VOPs can be reduced by around 20% at the cost of a higher absolute overestimation at high actual local SAR values.

**Conclusion:**

The proposed strategies outperform a previously published strategy and can improve the SAR compression where maximum relative overestimation constrains the performance of parallel transmission.

## Introduction

While single-channel and dual-channel transmit systems are still standard in clinical MRI systems, multi-channel parallel transmit (pTx) radiofrequency (RF) systems are often used at ultra-high field (UHF). Not only are these systems necessary to cope with the inhomogeneity introduced by the short wavelength of the RF signals [1, 2], these systems also offer more flexibility in excitation, especially at UHF [3, 4]. Examples of the techniques utilizing pTx systems are RF shimming [5, 6], kT-points [7], 2D spokes [8], 3D tailored radiofrequency pulses [9], Transmit SENSE [10, 11], and TIAMO [12]. Common among all these techniques is the use of arbitrary amplitudes and phases on the different transmit channels (excitation vector), whereby they differ in how rapidly the vectors are changed over time.

Altering the excitation vector changes the field patterns generated by the transmit system through its coil array including the E-fields in addition to the H-fields, and, therefore, the distribution of RF power absorption in the body tissue is changed as well. Regulatory guidelines [13] recommend constraints on specific absorption rate (SAR) averaged globally over the whole body (or, when appropriate, the region of the body exposed to RF fields), and averaged locally over any 10 g of tissue.

For a given combination of coil and subject on a single-channel system there is a fixed ratio between global SAR (SAR_global_) and maximum local SAR (SAR_max,local_), whereas in a pTx system there is no direct relation between the two. Furthermore, while in a single-channel system there is a direct relationship between input power and SAR, both SAR_global_ and SAR_local_ depend on the complex values of the excitation vector in a pTx system.

To ensure safety in pTx systems, numerical simulations are performed that use models of the arrays together with heterogeneous body models to calculate the fields inside the tissue [14]. These simulations provide maps of the local SAR that can be used to determine global and 10 g averaged SAR. This information can then be used for pulse design with SAR constraints as well as for online SAR supervision [15–17], but the number of voxels from such a simulation is very high and can reach orders of 10^6^.

To reduce the complexity of SAR calculation, the concept of virtual observation points (VOPs) [18] was introduced to compress the number of matrices. The general idea is to trade the number of voxels against an overestimation of the actual local SAR. Instead of calculating the SAR over all voxels, only a reduced number of VOPs need to be calculated. With this concept, the number of calculations necessary to approximate SAR_max,local_ can be reduced by several orders of magnitude. While the original clustering algorithm of Eichfelder et al. [18] is quite commonly used, a greedy algorithm was presented by Lee et al. [19] that achieves an even lower number of VOPs for a given problem and overestimation factor.

A drawback of both concepts is the fact that the overestimation is not (or at least not directly) dependent on SAR_max,local_. In the case of the Eichfelder algorithm, it is dependent on the worst-case SAR_max,local_ multiplied by a scalar_._ In the case of Lee, it is dependent on SAR_global_ multiplied by a scalar. This implies that the maximum absolute overestimation over all excitation vectors with unit power is fixed in relation to actual maximum local SAR for the Eichfelder algorithm and only slightly varying for the Lee algorithm. However, since the difference between the worst-case SAR_max,local_ and the lowest SAR_max,local_ can be more than an order of magnitude, the maximum relative overestimation can be significant. In practice this may lead to an unnecessary reduction of the duty cycle by a factor that is equivalent to the relative overestimation or a reduction of the allowed flip angle proportional to the square root of the relative overestimation.

For a typical array the worst-case SAR_max,local_ for a fixed input power is much higher than the lowest possible SAR_max,local_ for that same power, so the maximum relative overestimation is likely to be high, thus unnecessarily constraining array performance. This problem can be expected to be even more pronounced for arrays with a large numbers of coil elements, which can be illustrated by a simple example. In a close-fitting coil array, high local SAR can occur especially beneath the individual elements [20]. When all the power of a unit power excitation vector is transmitted via a single element, high local SAR will occur at this position, while a uniform distribution of power to all channels will lead to lower local SAR at least directly beneath the elements. The higher the number of channels, the more the power will be concentrated or distributed, respectively.

In this work, we present two ways of reducing maximum relative overestimation. The two compression schemes are both based on the greedy algorithm presented by Lee et al. [19]. The first one uses a matrix that roughly approximates the local SAR instead of using the global SAR matrix. The second one uses a set of VOPs to generate the necessary overestimation. These two new schemes are compared against Lee’s method.

## Materials and methods

### Overestimation with global SAR Matrix (“*S*_gobal_”)

Lee et al. define their VOP condition for the complete set $V_{{{\text{all}}}}$ of 10 g-averaged SAR matrices $S_{{v,10\;{\text{g}}}}$ by

1 $$\mathop {\max }\limits_{{v \in V_{{{\text{all}}}} }} \left\{ {b^{\prime}S_{{v,10\;{\text{g}}}} b} \right\} \le \mathop {\max }\limits_{{w \in V_{{{\text{sub}}}} }} \left\{ {b^{\prime}S_{{w,10\;{\text{g}}}} b} \right\} + \varepsilon_{{\text{G}}} b^{\prime}S_{{{\text{Global}}}} b\forall b \in {\mathbb{C}}^{{N_{{{\text{channel}}}} }}$$

Here $b$ is the excitation vector, $N_{{{\text{channel}}}}$ is the number of channels, $V_{{{\text{sub}}}} \subseteq V_{{{\text{all}}}}$ is a subset of the SAR matrices, $S_{{w,10\;{\text{g}}}}$ are the SAR matrices from the subset $V_{{{\text{sub}}}}$, $\varepsilon_{{\text{G}}}$ is the overestimation factor, and $S_{{{\text{Global}}}}$ is the global SAR matrix that calculates the global SAR from the input vector. We will call this strategy “*S*_global_” in the following parts of the manuscript, and it will serve as a reference standard for the two new algorithms. The rightmost term, $\varepsilon_{{\text{G}}} b^{\prime}S_{{{\text{Global}}}} b$, we call the overestimation term. In this work, we propose different strategies that use different overestimation terms.

### Overestimation with a constant factor (“*S*_diag_”)

Another strategy would be to use a constant overestimation term that is independent of the actual SAR. This can be achieved by replacing $S_{{{\text{Global}}}}$ in Eq. (1) by a matrix $S_{{{\text{diag}}}}$ that is a diagonal matrix with all elements of the main diagonal having the same value while all other values are zero. This is equivalent to using the spectral norm in Eq. (6) of Eichfelder’s paper. It has some similarity with the commonly used approach of using the Frobenius norm in Eq. (6) of said paper in the sense that the overestimation is independent of the actual SAR. The main difference is that due to the difference in the algorithms, Eichfelder’s iterative algorithm when using the Frobenius norm only adds overestimation in dimensions where it is necessary, while Lee’s algorithm adds the overestimation in all directions. The most notable difference being that the worst case SAR of an Eichfelder VOP set is the same the worst case SAR of the uncompressed set of SAR matrices, while the worst case SAR of a Lee VOP set is the worst case SAR of the uncompressed set of SAR matrices plus overestimation. In the following parts of the manuscript, we will call this strategy “*S*_diag_”, and it will serve as the second reference standard for the two new algorithms.

### Overestimation with approximated local SAR (“*S*_local_”)

Since the relation between $S_{{{\text{Global}}}}$ and local SAR cannot be expressed by means of a constant proportionality factor, the overestimation term in Eq. (1) only places an upper bound on the absolute overestimation error, but not the relative overestimation, which can lead to very large relative errors. To control the maximum relative overestimation, we propose using a matrix $S_{{{\text{local}}}}$ that is a rough approximation of local SAR:

2 $$\mathop {\max }\limits_{{v \in V_{{{\text{all}}}} }} \left\{ {b^{\prime}S_{{v,10\;{\text{g}}}} b} \right\} \le \mathop {\max }\limits_{{w \in V_{{{\text{sub}}}} }} \left\{ {b^{\prime}S_{{w,10\;{\text{g}}}} b} \right\} + \varepsilon_{{\text{G}}} b^{\prime}S_{{{\text{local}}}} b$$

Adding up all SAR matrices with appropriate weighting results in a matrix $S_{{{\text{Global}}}}$ that provides global SAR as described by Lee et al. [19], for example. To calculate a matrix $S_{{{\text{local}}}}$ that represents local SAR better, the weighting has to be changed. A simple approach for this is to calculate a VOP set and add up the VOPs, thereby putting more weight on those matrices that give a good representation of local SAR.

To calculate $S_{{{\text{local}}}}$ we use the original algorithm proposed by Lee to determine a subset $V_{{\text{sub,pre}}} { \subsetneq }V_{{{\text{all,1\% }}}} { \subsetneq }V_{{{\text{all}}}}$, where we reduce the complexity of the calculation by first randomly selecting a subset of only 1% of all SAR matrices ($V_{{{\text{all}},1\% }}$) to which we apply the Lee algorithm. We then sum up the matrices of the calculated sub volume $V_{{\text{sub,pre}}}$ (VOPs) to get a matrix $S_{{{\text{pre}}}}$ that has some properties of local SAR.

3 $$S_{{{\text{pre}}}} = \mathop \sum \limits_{{v \in V_{{{\text{sub}},{\text{pre}}}} }} S_{{v,10\;{\text{g}}}}$$

To achieve an even better approximation of local SAR, we calculate the eigenvectors of $S_{{{\text{pre}}}}$ and calculate the local SAR for these eigenvectors from the full set of SAR matrices. $S_{{{\text{local}}}}$ is then defined as follows:

4 $$S_{{{\text{local}}}} = VDV^{\prime},$$

where $V$ is a matrix whose columns are the eigenvectors and $D$ is a diagonal matrix where the diagonal element $d_{ii}$ is the SAR value ${\text{SAR}}_{{\text{maxlocal eig i}}}$ corresponding to the *i*th eigenvector. Since the difference between the highest and the lowest SAR value in $D$ can be quite large and lead to extreme differences in the overestimation, we suggest using an exponential scaling term $R \ge 1$ to reduce the ratio and empirically find a matrix that provides good results (low maximum relative overestimation) in the compression:

5 $$d_{ii} = {\text{SAR}}_{{\text{maxlocal eig i}}}^{\frac{1}{R}}$$

We suggest to use an $R$ so that the ratio between the maximum and the minimum eigenvalue of $S_{{{\text{local}}}}$ is below 10.

In the following parts of the manuscript, we will call this strategy “*S*_local_”, and it is one of the two new algorithms we propose to control maximum relative overestimation.

### Overestimation by pre calculated VOPs (“Double VOP”)

While the above approach of using a single overestimation matrix to approximate local SAR for the overestimation term is simple in that after calculating $S_{{{\text{local}}}}$ the algorithm as defined in [19] can be used, it has the drawback that $S_{{{\text{local}}}}$ only provides a very rough correlation with local SAR. To get a better correlation, we can use the maximum local SAR value calculated from a small pre-calculated set of VOPs $V_{{{\text{sub}},{\text{pre}}}}$ for the overestimation:

6 $$\mathop {\max }\limits_{{v \in V_{{{\text{all}}}} }} \left\{ {b^{\prime}S_{{v,10\;{\text{g}}}} b} \right\} \le \mathop {\max }\limits_{{w \in V_{{{\text{sub}}}} }} \left\{ {b^{\prime}S_{{w,10\;{\text{g}}}} b} \right\} + \varepsilon_{{\text{G}}} \mathop {\max }\limits_{{u \in V_{{{\text{sub}},{\text{pre}}}} }} \left\{ {b^{\prime}S_{{u,10\;{\text{g}}}} b + \varepsilon_{{{\text{G}},{\text{pre}}}} b^{\prime}S_{{{\text{Global}}}} b} \right\}$$

Here $\varepsilon_{{{\text{G}},{\text{pre}}}}$ is the overestimation factor used to calculate the set of pre-calculated VOPs $V_{{{\text{sub}},{\text{pre}}}}$. This set can be calculated by using the algorithm proposed by Lee et al. The scaling factor $\varepsilon_{{\text{G}}}$ can be used to scale the maximum overestimation.

At first, it might appear that Eq. (6) is similar to

7 $$\mathop {\max }\limits_{{v \in V_{{{\text{all}}}} }} \left\{ {b^{\prime}S_{{v,10\;{\text{g}}}} b} \right\} \le \left( {1 + \varepsilon_{{\text{G}}} } \right)\mathop {\max }\limits_{{w \in V_{{{\text{sub}}}} }} \left\{ {b^{\prime}S_{{w,10\;{\text{g}}}} b} \right\},$$

i.e., using a simple scaling factor, but during compression these two equations are obviously not equivalent. By looking at the very first step of the greedy algorithm, this becomes clear. In the first step the matrix with the highest eigenvalue is compared to the matrix with the second highest eigenvalue. In the case of Eq. (6) a term is added containing information on the maximum local SAR over all voxels (plus an overestimation term); in the case of Eq. (7) only the information from the very first matrix is used. Over the course of the algorithm, this leads to many more voxels being included in the subset.

When applying Eq. (6), the algorithm proposed by Lee et al. [19] has to be modified slightly. First we replace the condition in Eq. (6) by its identical condition in terms of matrix inequalities [21, 22], equivalent to how it was done in [19]:

8 $$S_{{v,10\;{\text{g}}}} \le \mathop \sum \limits_{{w \in V_{{{\text{sub}}}} }} c_{w,v} S_{{w,10\;{\text{g}}}} + \varepsilon_{{\text{G}}} \mathop \sum \limits_{{u \in V_{{{\text{pre}}}} }} c_{u,v} \left( { S_{{u,10\;{\text{g}}}} + \varepsilon_{{{\text{G}},{\text{pre}}}} S_{{{\text{Global}}}} } \right)$$

In each greedy step of the algorithm, the iterative method from Lee et al. [19] is used, with modifications necessary to incorporate the changed terms:

1. Initialization of the two sets of coefficients $c_{w,v}$ and $c_{u,v}$. In each set of coefficients, the coefficients are set to equal values with an L1 norm of 1.
2. If all the eigenvalues of the matrix $P = \mathop \sum \limits_{{w \in V_{{{\text{sub}}}} }} c_{w,v} S_{{w,10\;{\text{g}}}} + \varepsilon_{{\text{G}}} \mathop \sum \limits_{{u \in V_{{{\text{pre}}}} }} c_{u,v} \left( { S_{{u,10\;{\text{g}}}} + \varepsilon_{{{\text{G}},{\text{pre}}}} S_{{{\text{Global}}}} } \right) - S_{{v,10\;{\text{g}}}}$ are nonnegative, the voxel $v$ can be upper-bounded by the previously determined VOPs.
3. If not, calculate the eigenvector $b$ of $P$ corresponding to the minimum eigenvalue of $P$. If $b^{\prime}S_{{v,10\;{\text{g}}}} b > \max_{{w \in V_{{{\text{sub}}}} }} \left\{ {b^{\prime}S_{{w,10\;{\text{g}}}} b} \right\} + \varepsilon_{{\text{G}}} \max_{{u \in V_{{{\text{pre}}}} }} \left\{ {b^{\prime}S_{{u,10\;{\text{g}}}} b + \varepsilon_{{{\text{G}},{\text{pre}}}} S_{{{\text{Global}}}} } \right\}$, the voxel $v$ cannot be upper-bounded and is included in the subset.
4. If neither the condition from step 2 nor from step 3 is satisfied, update the coefficients to make $b^{\prime}{\text{Pb}}$ nonnegative:
5. Calculate $$m = b^{\prime}{\text{Pb}}$$
6. a. Randomly change one coefficient of $c_{w,v}$ and normalize so that the L1 norm remains 1. Calculate $m_{{{\text{temp}}}} = b^{\prime}{\text{Pb}}$. If $m_{{{\text{temp}}}}$ is nonnegative, go to step 5. If $m_{{{\text{temp}}}}$ is smaller than $m$, undo the coefficient change, else set $m = m_{{{\text{temp}}}}$
7. b. Randomly change one coefficient of $c_{u,v}$ and normalize so that the L1 norm remains 1 and calculate $m_{{{\text{temp}}}} = b^{\prime}{\text{Pb}}$. If $m_{{{\text{temp}}}}$ is nonnegative, go to step 5. If $m_{{{\text{temp}}}}$ is smaller than $m$, undo the coefficient change, else set $m = m_{{{\text{temp}}}}$
8. c. Repeat
9. Iterate step 2–4. If any of the conditions are not satisfied by the time the number of iterations exceeds a pre-selected maximum, add the voxel to $V_{{{\text{sub}}}}$.

This algorithm results in two sets of VOPs. The maximum of the pre-calculated set is multiplied by $\varepsilon_{{\text{G}}}$ and added to each SAR value of the newly calculated set, then these values can be used just like the results of the other algorithms. In the following parts of the manuscript, we will call this strategy “Double VOP”, and it is the second of the two new algorithms we propose to control maximum relative overestimation.

### Coil arrays

**Fig. 1:**
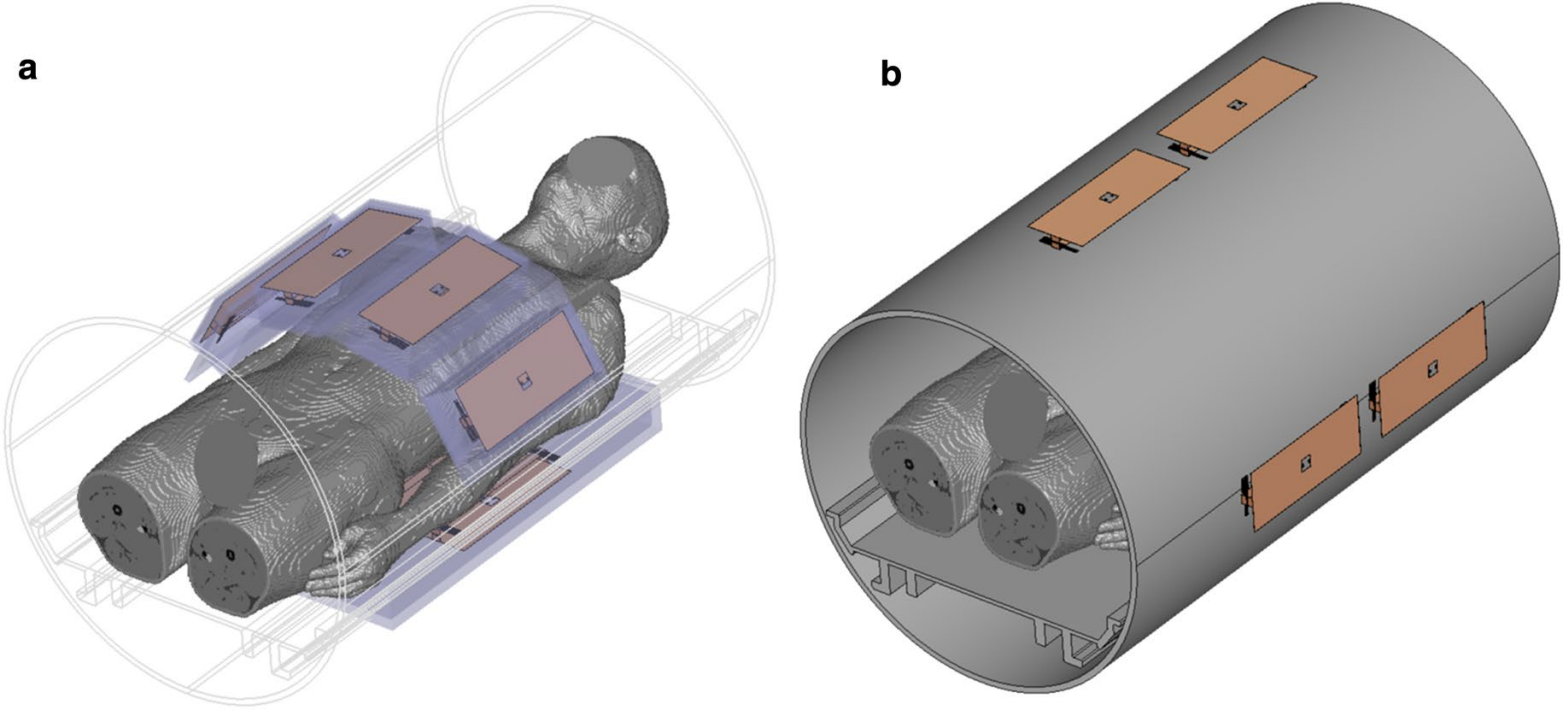
Coil models used in this study. **a** Shows the flexible local body array and **b** shows the remote array. The simulation models contain the housing of the coil (where applicable), the patient table, the bore liner, and the magnet cryostat (not shown)

To test the algorithms, the SAR matrices of two different eight-channel arrays made from micro strip lines with meanders [23] and operating at the proton resonance frequency of 7 T were used. The first array is a local array placed directly on the body [24], while the second array is a remote array in 2 × 4 configuration positioned behind the bore liner (Fig. 1). All simulations were performed in CST Microwave Studio 2017 (CST AG, Darmstadt, Germany) and considered the MR environment (patient table, bore liner, gradient coil, and cryostat) as well as the coil housing where applicable.

The local array was tuned to resonance and matched to 50 Ohms using a capacitor network in a co-simulation. The 2 × 4 remote array configuration was ideally tuned and decoupled by applying a decoupling matrix consisting of lossless inductors and capacitors that interconnects the transmit elements [25, 26]. No decoupling matrix was applied to the local array.

A heterogeneous body model (male, 174 cm, 70 kg, tissue resolution 2 × 2 × 2 mm^3^) [27] in head-first supine position with the liver–kidney region in the center was used for both arrays. The simulation domain was discretized with approximately 65 million mesh cells. Matrices for 10 g-averaged local SAR were calculated for both setups resulting in 7.6 million matrices for the local array and 6.5 million matrices for the remote array.

### Algorithm implementation and calculation of results

The algorithms were implemented in Matlab (The Mathworks Inc., Natick, MA, USA) with a high degree of vectorization and other optimizations to speed up the calculations. The software developed for this paper is available as open source at sourceforge.net (https://sourceforge.net/projects/relative-overestimation-vop/). All compressions were performed on a PC with two 6-core Xeon X5690 processors (Intel, Santa Clara, CA, USA) with 128 GB of 1333 MHz DDR3 RAM.

1. The original dataset was compressed in the four ways explained above:
  With the global SAR matrix (*S*_global_) according to Lee et al. [19] as the first reference standard
2. With a diagonal matrix (*S*_diag_) where all values on the main diagonal are equal to the worst-case local SAR, leading to an overestimation that is independent of actual local SAR as the second reference standard
3. With the new approach using a matrix approximating local SAR (*S*_local_)
4. with the new approach utilizing a set of pre-calculated VOPs to define the maximum overestimation (Double VOP). The value of $\varepsilon_{{{\text{G}},{\text{pre}}}}$ was chosen to obtain 10 or fewer pre-calculated VOPs.

Furthermore, a compression with the Eichfelder algorithm was performed for comparison.

The compressions for all different strategies were repeated with different factors $\varepsilon_{{\text{G}}}$ to obtain datasets with different numbers of VOPs. The SAR results obtained with these VOPs were then compared to the results of the uncompressed dataset for 1 million random excitation vectors to find the maximum relative overestimation.

## Results

The pre-calculations for the Double VOP approach took less than 10 min and resulted in ten pre-calculated VOPs for the local array ($\varepsilon_{{{\text{G}},{\text{pre}}}} = 0.2)$ and nine pre-calculated VOPs for the remote array $(\varepsilon_{{{\text{G}},{\text{pre}}}} = 0.1)$.

**Fig. 2:**
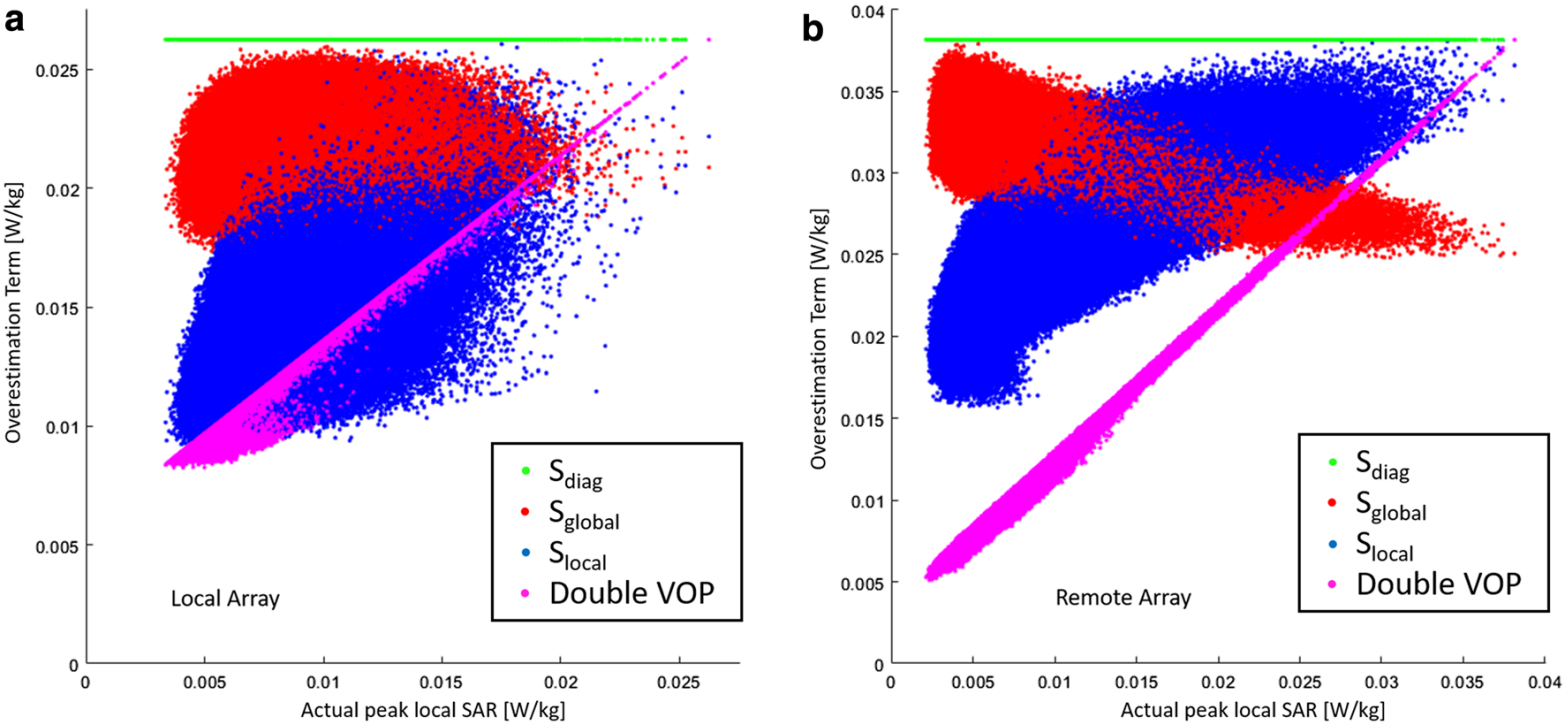
Comparison of the value of the overestimation term for the different strategies for **a** the local array and **b** the remote array for a set of 1 million random excitation vectors with unit power. The *x*-axis shows the actual maximum local SAR calculated with the uncompressed set of SAR matrices; the* y*-axis shows the result of the overestimation term resulting from the respective excitation vector. Each overestimation term was normalized so that its maximum corresponds to worst-case actual local SAR

The overestimation terms from Eqs. (1), (2), and (6) were compared to the actual SAR value calculated with the uncompressed dataset and 1 million random excitation vectors. For readability, the values were normalized so that their maximum values are equal to the worst-case actual local SAR for unit power excitation. The results for both array configurations are shown in Fig. 2. Figure 2a shows the results for the local array. While the results for *S*_diag_ and *S*_global_ are more or less uncorrelated with the actual local SAR, the overestimation terms for *S*_local_ and especially Double VOP are lower when actual local SAR is lower.

In the case of the remote array, there is a visible negative correlation between the overestimation term of *S*_global_ and the actual SAR (Fig. 2b). For low actual SAR values, the overestimation term is larger than it is for higher local SAR values, leading to a potentially higher relative overestimation at low SAR values. The overestimation terms for *S*_local_ and Double VOP show a visible positive correlation with actual local SAR, resulting in smaller relative overestimation at low SAR values.

**Fig. 3:**
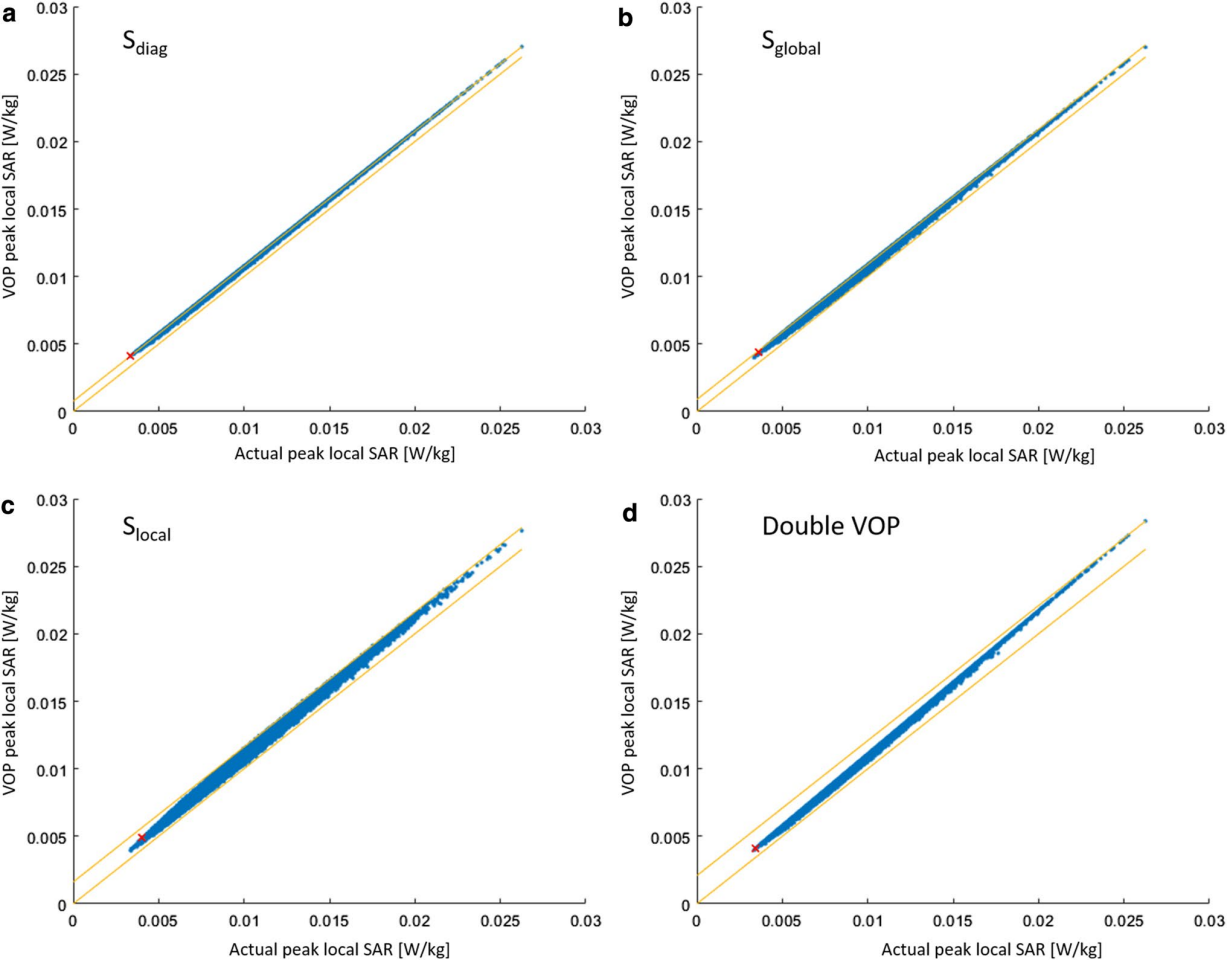
Example results for the local array. The *x*-axis shows the actual local SAR calculated from the uncompressed dataset, while the *y*-axis shows the result of the respective compressed dataset with around 100 VOPs. A red cross marks the position of the maximum relative overestimation in each figure. The yellow lines denote the upper and lower bound (maximum absolute overestimation and zero overestimation). Note that for Double VOP and *S*_local_, the values of VOP SAR adapt to the lower bound for low actual SAR values

**Fig. 4:**
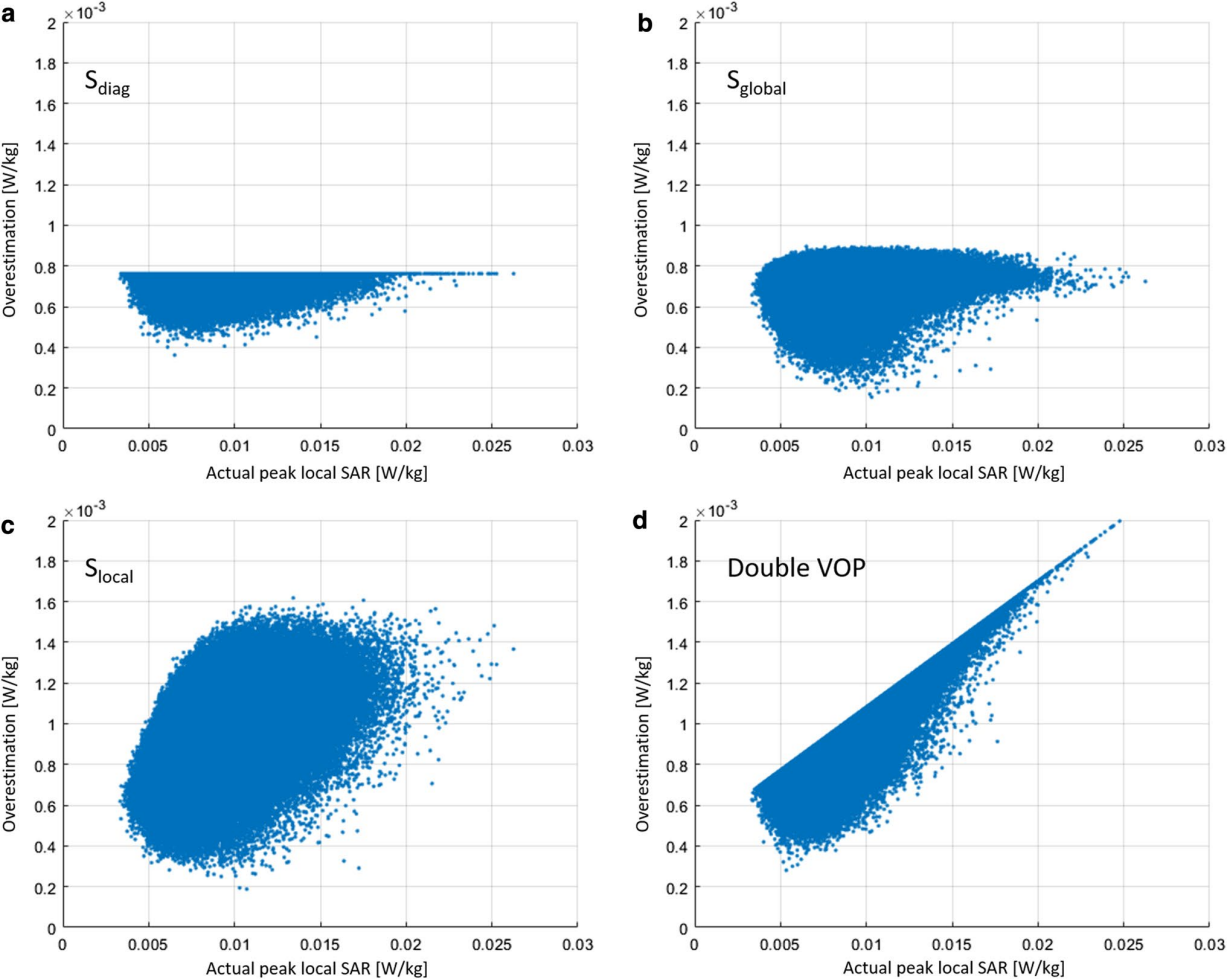
Absolute overestimation of the VOP-calculated SAR versus the actual SAR for the local array. Each respective set contains a total of around 100 VOPs

Example results in Fig. 3 show the VOP-calculated maximum local SAR versus the actual maximum local SAR in the local coil configuration for *S*_diag_ (a), *S*_global_ (b), *S*_local_ (c), and Double VOP (d), while Fig. 4 shows the absolute overestimation. The number of VOPs for all cases was approximately 100, corresponding to a minimum overestimation term of around 2% of the worst case local SAR. Red crosses denote the position of the maximum relative overestimation; the yellow line is the unity line, included for reference. First, it can be noted that no underestimation occurred, which was to be expected since the mathematical concept has already been proved to be correct by Lee et al. [19]. It can be seen that the four different overestimation terms result in different actual overestimations. Using *S*_diag_ the maximum absolute overestimation for a given actual SAR value is constant versus the actual SAR, which can also be appreciated in Fig. 4a, while in the other three cases it shows some dependence on the actual local SAR. *S*_local_ shows a reduction in the absolute overestimation below approximately 0.0075 W/kg (Fig. 4c), while Double VOP (Fig. 4d) shows a linear behavior as could be expected from the results shown in Fig. 2a. The calculation time for the cases with a single matrix for the overestimation was approximately 90 min each, while the double VOP approach took 110 min. For comparison, the Eichfelder algorithm needed 12 min for the 100 VOPs, while it took 55 min for the overestimation of 2% of worst-case SAR and produced 856 VOPs.

**Fig. 5:**
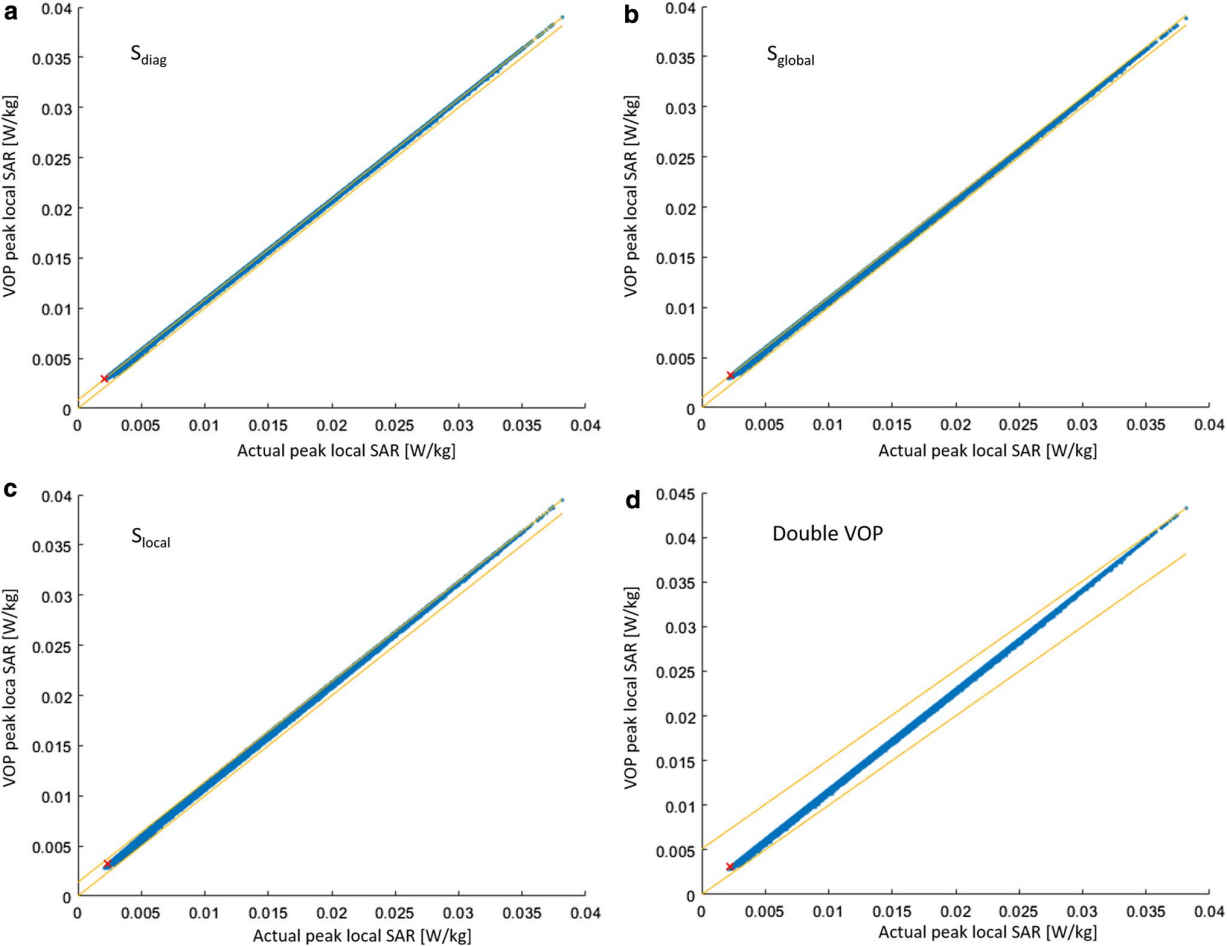
Example results for the remote array. The x-axis shows the actual local SAR calculated from the uncompressed dataset, while the y-axis shows the result of the respective compressed dataset with around 45 VOPs. A red cross marks the position of the maximum relative overestimation in each figure. The yellow lines denote the upper and lower bound (maximum absolute overestimation and zero overestimation). Note that for Double VOP and *S*_local_, the values of VOP SAR adapt to the lower bound for low actual SAR values

**Fig. 6:**
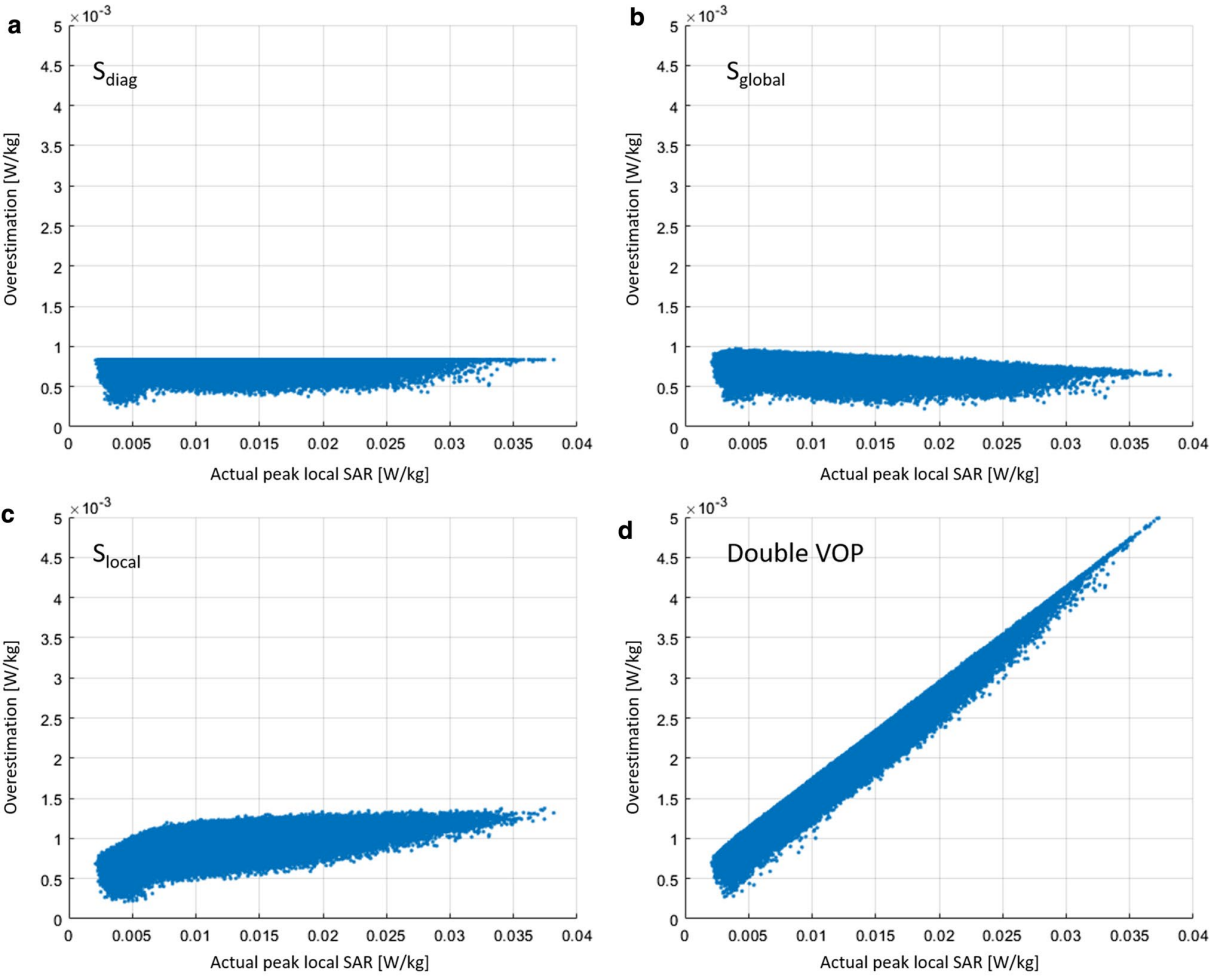
Absolute overestimation of the VOP-calculated SAR versus the actual SAR for the remote array. Each respective set contains a total of around 45 VOPs

Example results for the remote array are given in Fig. 5. Again, the VOP-calculated maximum local SAR versus the actual local SAR in the local coil configuration for *S*_diag_ (a), *S*_global_ (b), *S*_local_ (c), and Double VOP (d) is shown, while Fig. 6 provides the corresponding absolute overestimation. Here, the number of VOPs was around 45 for all cases, corresponding to a minimum overestimation term of around 2% of the worst-case local SAR. It can be noted that the ratio of the maximum actual SAR over the minimum actual SAR is significantly larger than for the case of the local array. The absolute overestimation using *S*_global_ is larger for small actual SAR values than for large SAR values, significantly increasing the relative overestimation for small SAR values. Using *S*_local_ and Double VOP, a smaller absolute overestimation occurs at lower actual SAR values. The calculation time for the cases with a single matrix for the overestimation was approximately 50 min each, while the double VOP approach took 70 min. For comparison, the Eichfelder algorithm needed 12 min for the 45 VOPs, while it took 55 min for the overestimation of 2% of worst-case SAR and produced 330 VOPs.

**Fig. 7:**
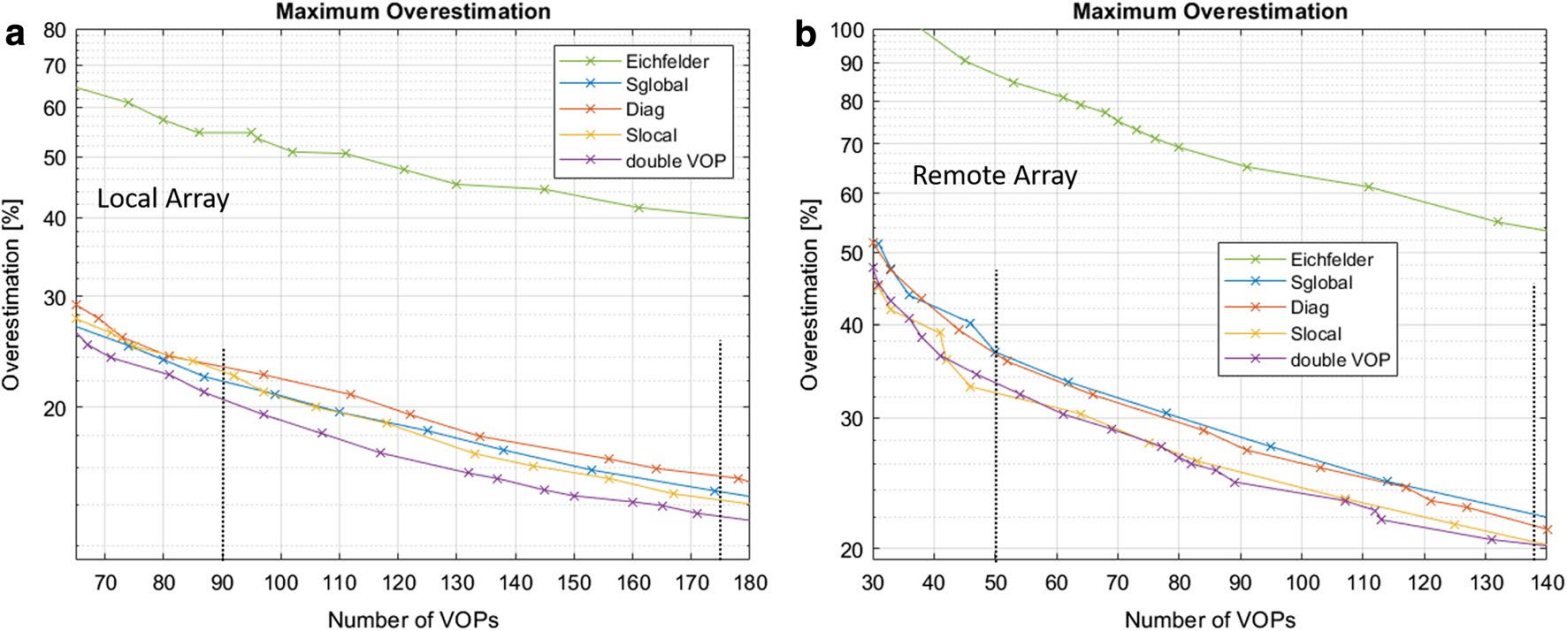
Maximum relative overestimation versus number of VOPs. The crosses denote the calculated results; the connecting lines are included for better readability. Logarithmic scaling is used to include the results for the algorithm proposed by Eichfelder et al. In the areas between the dashed black lines, the mean reduction in the number of VOPs is 17% for the local array and 24.6% for the remote array, respectively

A direct comparison for all compression strategies is shown in Fig. 7. The figure shows the maximum relative overestimation in percent for the local array (7a) and remote array (7b). Crosses denote the results obtained from compression; the connecting lines are included to improve readability. Note that for the Double VOP strategy, the number of VOPs is the number of VOPs from the compression plus the number of pre-calculated VOPs. As is to be expected, a larger number of VOPs results in a smaller maximum relative overestimation. In both configurations, Double VOP offers the best result, while *S*_local_ outperforms *S*_global_ in the remote array configuration but provides equivalent results in the local array configuration. In the remote array configuration, *S*_global_ provides the overall worst result, being outperformed even by the simple *S*_diag_.

In the areas between the dashed black lines in Fig. 7, the mean reduction in the number of VOPs is 17% for the local array and 24.6% for the remote array, respectively.

## Discussion

In this work we show results with up to 180 VOPs for the Lee algorithm. This is close to the maximum number of 200 VOPs provided by Jin et al. as the maximum number that the pulse supervision of their vendor provided system can handle [28]. Due to the difference in compression efficiency, this number corresponds to roughly 1000 VOPs calculated by the Eichfelder algorithm when applying the same overestimation. The maximum number of VOPs that can be handled by the supervision system will depend on the particular hardware and software implementation as well on the number of elements in the transmit array.

The results for the overestimation terms show that *S*_global_ is not necessarily a good choice. As shown here for the remote array, there are cases in which *S*_global_ leads to an increased absolute overestimation at lower actual SAR values, leading to a disproportionately high relative overestimation. In this case, even a fixed overestimation (*S*_diag_) is preferable. Using an overestimation term that approximates actual local SAR (*S*_local_) proved preferable in this work, since it was at least as good as using *S*_global_. The *S*_local_ approach does not require any changes to the pulse calculation or pulse supervision software.

The overall best results for maximum relative overestimation were obtained using the Double VOP approach. A downside of this approach is the fact that the set of VOPs after compression is not directly compatible with previous algorithms for pulse calculation or supervision, since the results for two separate sets of VOPs have to be calculated and subsequently added up. Therefore, using this approach necessitates some software adaptations. While the implementation in many pulse calculation algorithms might be tricky because of having to implement the maximum of the VOP set used to calculate the overestimation term, the implementation into SAR supervision is straightforward. The calculation effort when calculating *N*_VOPs_ VOPs in addition to *N*_VOPs,pre_ pre-calculated VOPs should be almost the same as using the same number VOPs in the standard algorithm: *N*_VOPs,standard_ = *N*_VOPs _+ *N*_VOPs,pre_. In this case, only *N*_VOPs_ more addition operations are necessary in comparison to the standard method, which is not much effort considering that calculating the SAR for a single VOP in an eight-channel configuration takes 63 addition operations and 72 multiplication operations.

Using the proposed strategies *S*_local_ and Double VOP, the number of VOPs could be reduced by around 20% while obtaining the same maximum relative overestimation in comparison to the original Lee algorithm. Overall, the two presented strategies provide a trade-off between maximum relative overestimation and maximum absolute overestimation.

## Conclusion

In this paper, we present two strategies to reduce maximum relative overestimation in VOP compression. Both strategies are able to reduce the maximum relative overestimation for a fixed number of VOPs. Furthermore, we show that using global SAR for the overestimation term is not necessarily a good choice. Using the strategies proposed in this paper can lead to enhanced performance of VOP compressions and therefore more exact and/or faster calculation of local SAR. Practically, this implies improved performance of multi-channel RF arrays in pTx applications.
